# No Evidence of Metabolic Depression in Western Alaskan Juvenile Steller Sea Lions (*Eumetopias jubatus*)

**DOI:** 10.1371/journal.pone.0085339

**Published:** 2014-01-09

**Authors:** Lisa A. Hoopes, Lorrie D. Rea, Aaron Christ, Graham A. J. Worthy

**Affiliations:** 1 Department of Wildlife and Fisheries Sciences, Texas A&M University, College Station, Texas, United States of America; 2 Alaska Department of Fish and Game, Division of Wildlife Conservation, Anchorage, Alaska, United States of America; 3 Department of Biology, University of Central Florida, Orlando, Florida, United States of America; Clemson University, United States of America

## Abstract

Steller sea lion (*Eumetopias jubatus*) populations have undergone precipitous declines through their western Alaskan range over the last four decades with the leading hypothesis to explain this decline centering around changing prey quality, quantity, or availability for this species (i.e., nutritional stress hypothesis). Under chronic conditions of reduced food intake sea lions would conserve energy by limiting energy expenditures through lowering of metabolic rate known as metabolic depression. To examine the potential for nutritional stress, resting metabolic rate (RMR) and body composition were measured in free-ranging juvenile Steller sea lions (*N* = 91) at three distinct geographical locations (Southeast Alaska, Prince William Sound, Central Aleutian Islands) using open-flow respirometry and deuterium isotope dilution, respectively. Average sea lion RMR ranged from 6.7 to 36.2 MJ d^−1^ and was influenced by body mass, total body lipid, and to a lesser extent, ambient air temperature and age. Sea lion pups captured in the Aleutian Islands (region of decline) had significantly greater body mass and total body lipid stores when compared to pups from Prince William Sound (region of decline) and Southeast Alaska (stable region). Along with evidence of robust body condition in Aleutian Island pups, no definitive differences were detected in RMR between sea lions sampled between eastern and western populations that could not be accounted for by higher percent total body lipid content, suggesting that that at the time of this study, Steller sea lions were not experiencing metabolic depression in the locations studied.

## Introduction

Juvenile pinnipeds experience unique energetic challenges early in life that can impact individual survival and even species population dynamics (e.g., [1] , [2]). Compared to adults, juveniles must balance the added energetic expense associated with an elevated metabolism and the heat production associated with the biochemical synthesis of tissue for growth with increased thermoregulatory costs given the high surface to volume ratio associated with their small size [3]. While this is certainly a concern for juvenile pinnipeds on land at high latitudes, all pinnipeds must eventually enter the water to forage where the potential for conductive and convective heat loss is considerably higher. The transition from weaning to independence is a critical factor determining survival in juvenile pinnipeds (e.g., [4]). Foraging inexperience combined with reduced diving capabilities (e.g., smaller total oxygen stores, high metabolic rates) can make prey resources difficult to access while exposing young animals to extended periods of elevated heat loss while searching for food. The challenge of maintaining energetic balance in juvenile pinnipeds is further magnified when resource availability, such as access to prey, becomes unpredictable or scarce.

Under conditions of reduced food availability juvenile pinnipeds would be faced with two divergent options; they can either increase foraging effort or limit energy expenditures in order to maintain energy balance. Increased foraging effort is a short-term gamble for the animal in that additional energy has to be expended on the front end which will be off-set in the event that prey is encountered while foraging [5]. Limiting energy expenditures is a longer-term strategy used by animals during predictable or prolonged shortages in energy intake [6], [7]. The criterion in which an animal chooses one strategy over the other is not well understood.

Animals can limit energy expenditures through decreased activity, increased sleep, and/or a lowering of core body temperature and metabolism. A common physiological response to undernutrition or fasting is to lower basal metabolic rate, known as metabolic depression. Adaptation to periods of food restriction requires not only adjustments in metabolism, but also in fat deposition, in hormonal regulation, and in mobilization of fuel reserves in order to slow tissue loss and ultimately extend the time an organism can survive (e.g., [8]). Metabolic depression as a response to environmental stress has been observed in nearly all major animal taxa [9] including several species of marine mammals [10]–[18], [6].

Metabolic depression typically occurs during natural periods of prolonged fasting usually associated with weaning and molting in phocid pinnipeds [11], [14]–[16], [19]. Although adult otariids sustain short periods of fasting associated with breeding and pup rearing, juvenile otariids generally do not undergo extensive periods of fasting associated with changes in life history. However, this age class could experience periods of reduced intake associated with unpredictability in prey availability over spatial and temporal scales. Diet restriction studies with captive Steller sea lions showed reductions in metabolic rate (30%) when animals were fasted for 9–14 d, however, this decrease in metabolic rate was not sufficient to prevent body mass loss [20], [6].

Steller sea lion populations have shown precipitous declines starting in the late 1970’s throughout their western Alaskan range (Aleutian Islands and Gulf of Alaska) [2], while numbers in Southeastern Alaska, British Columbia and Oregon have remained stable or slightly increased [21]. The greatest population declines since the 1970’s occurred in the eastern Aleutian Islands and western Gulf of Alaska. More recently, western U.S. Steller sea lion stocks decreased 40% from 1991–2000, with an average annual decline of about 5.4% [22]. Survey data suggested that from 2000–2004, non-pup sea lion counts had stabilized or increased slightly in the eastern Aleutian Islands and central and western Gulf of Alaska, although there was considerable regional variability and population levels continued to decline in both the central and western Aleutian Islands [23]–[25]. While a number of hypotheses have been proposed to explain the rapid decline of western Alaskan Steller sea lions, the leading hypothesis was that these populations experienced either a reduction in overall prey abundance or change in relative abundance of the type and quality of prey available [26]. Reductions in prey abundance would be particularly challenging to juvenile animals, thus potentially reducing recruitment of this life history stage into the breeding population (e.g., [2]).

If Steller sea lions were continuing to decline in western Alaskan populations due to reductions in prey abundance (i.e., nutritional stress hypothesis) then we would expect to see evidence of this reflected in energy maintenance requirements among sea lions from the different regions, with metabolic depression evident in areas of possible food limitation. The goal of the present study was to measure metabolic rate in free-ranging juvenile sea lions with regard to ontogeny, ambient temperature, and body condition; with the primary objective of determining if animals from the declining western population showed evidence of metabolic depression.

## Materials and Methods

### Ethics Statement

All sea lion capture, handling, and research was conducted under Marine Mammal Protection Act (MMPA) permit number 358-1564 issued to the Alaska Department of Fish & Game (ADF&G). Animal use protocols used in this research were reviewed and approved by the University Laboratory Animal Care Committee at Texas A&M University (protocol 2001-319) and the Institutional Animal Care and Use Committee at the State of Alaska Department of Fish & Game (protocol 03-0002).

### Study Area and Sea Lion Capture and Handling

**Figure 1:**
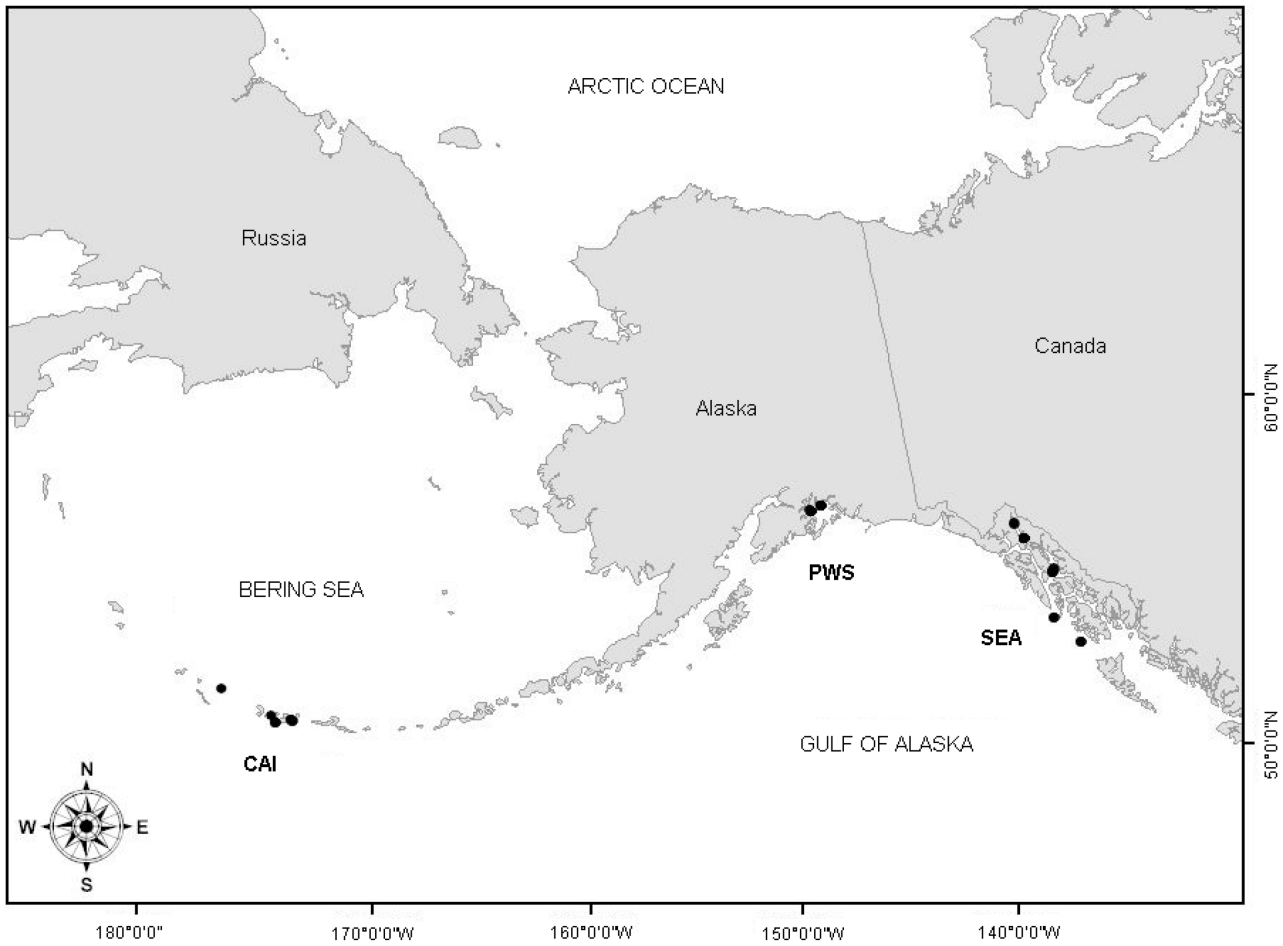
Map of Alaska showing capture locations (black circles) within each of the three sampling regions. The Central Aleutian Islands (CAI) and Prince William Sound (PWS) are within declining western DPS and Southeast Alaska (SEA) is within the stable eastern DPS.

Ninety-one juvenile Steller sea lions ranging in age from 2 to 44 months were captured throughout their Alaskan range between 2003 and 2005 (Table 1). Individual animals originated from three distinct geographical regions: (1) Southeast Alaska (SEA, threatened eastern distinct population segment (DPS)) ranging from the southern border of Alaska north to Cape Suckling, (2) Prince William Sound (PWS, endangered western DPS), and (3) the Central Aleutian Islands (CAI, endangered western DPS) (Figure 1). Within each region, sea lions were captured in the waters surrounding known haul out or rookery locations using SCUBA divers employing an underwater capture technique developed by the ADF&G [27]. Occasionally sea lions were captured on land with hoop nets when weather or current conditions prevented in-water capture.

**Table 1:** Mean (± SE) body mass, resting metabolic rate, and total body lipid for sea lions captured for this study.

Location	Sample Date	Age (mo)	N	Body Mass (kg)	RMR (MJ d^−1^)	TBL (%)
CAI	Apr-05	10.5	16	130.2 ± 7.1	21.0 ± 1.5	32.9 ± 1.4
PWS	Nov-03	5	15	77.1 ± 3.7	21.0 ± 1.3	22.6 ± 1.0
	Feb-05	8	3	94.7 ± 4.7	15.0 ± 2.7	27.4 ± 3.8
	Nov-03	17	8	132.5 ± 4.7	28.3 ± 0.9	20.8 ± 3.2
	Feb-05	20	3	150.5 ± 13.4	23.0 ± 2.6	21.7 ± 3.2
	Feb-05	44	1	232.5	29.2	13.5
SEA	Aug-04	2	9	38.8 ± 1.5	11.0 ± 1.1	12.8 ± 1.9
	Feb-04	8	18	78.8 ± 3.3	16.4 ± 0.8	27.7 ± 1.9
	Aug-04	14	9	101 ± 4.9	20.6 ± 0.6	18.6 ± 1.4
	Feb-04	20	5	127.4 ± 6.4	20.1 ± 1.2	30.2 ± 2.2
	Aug-04	26	4	144.6 ± 7.0	32.3 ± 1.3	13.8 ± 1.5

N, sample size; RMR, resting metabolic rate; TBL, total body lipid; CAI, Central Aleutian Islands; PWS, Prince William Sound; SEA, Southeast Alaska.

Approximately 1–2 h post-capture, sea lions were weighed (Ocean King D-6, TCI Scales, Inc., Mukilteo, WA, ±0.5 kg) in their capture boxes and then immobilized under gas anesthesia based on methods detailed by Heath et al. [28]. Age and sex were determined. With the exception of the youngest pups whose age was estimated by average pupping date (June 15, [29]), age was estimated using date, body size, and degree of tooth eruption or canine length [30]. Animals were intubated to collect gastric contents in order to determine recent nursing or feeding activity.

### Metabolic Rate

Resting metabolic rate (RMR) during all trials was measured under ambient air temperature conditions using open-flow respirometry. Air temperature averaged 3.2 °C (range 1.3 to 5.1°C) in AI, –0.6 °C (range –6.5 to 3.7°C) in PWS, and 10.2°C (range 2.9 to 19.2°C) in SEA. Sea lions held in metal capture boxes with insulating padding were placed on the wooden base of the metabolic chamber, over which a Plexiglas® lid (231×79×56 cm) was fitted into metal tracking. An airtight seal between the lid and base was created by filling the track initially with seawater, and then in subsequent trials with foam-rubber. Fans were mounted in each of the four corners of the lid to promote continuous air mixing and internal chamber temperature was continuously monitored by a thermocouple (Weather Monitor II, Davis Instruments, CA, USA). Under sunny summer conditions, tarps were erected over the chamber to prevent chamber temperatures from getting too warm. Sea lion behavior was closely monitored along with chamber temperature, and trials were immediately ended if chamber temperatures approached 24 °C or if sea lions exhibited signs of heat stress (e.g., open mouth breathing).

Ambient air was drawn through the chamber at a constant rate (70–200 l min^−1^, based on animal size) with flow rates maintained and monitored with a mass flow controller (Flowkit 500, Sable Systems International, NV, USA). A subsample of air was dried (Drierite, W.A. Hammond, OH, USA) and scrubbed of carbon dioxide (Sodasorb, Chemetron, MO, USA) before entering an oxygen gas analyzer (FC-1B, Sable Systems, NV, USA) and a carbon dioxide gas analyzer (CA-2A, Sable Systems, NV, USA), respectively. Gas analyzers were calibrated before and after a research trip with dry ambient air (20.94% O_2_) and 1.0% CO_2_ in N_2_ gas according to Fedak et al. [31]. Oxygen consumption (VO_2_) and carbon dioxide production (VCO_2_) measurements were recorded for a minimum of 1 h, and up to 2 h in duration. The lowest VO_2_ measurements maintained for a continuous 15 min period and corresponding to quiescent behavior and stable chamber temperatures were used for the analyses. VO_2_ and VCO_2_ measurements were used to calculate RMR based on standard equations by Withers [32] and were converted to energy utilization assuming that 1 l O_2_  =  20.0 kJ.

RMR was measured on animals post-anesthesia. To determine the potential effects of anesthesia on metabolism, VO_2_ was measured before and after waking from anesthesia in a subset of animals (*N* = 19), varying in age from 5 to 20 months. All sea lions were awake when placed in the chamber. Sea lion activity was monitored and recorded every 5 min, or whenever activity levels changed, and tapping on the chamber prevented animals from sleeping. For discussion purposes, sea lions < 12 months in age were considered pups, while animals ≥ 12 months of age were considered juveniles.

### Body Condition

Body condition was assessed as part of a separate, concurrent study by measuring the thickness of the subcutaneous blubber layer (*N* = 87) and by estimating body composition (proportion of adipose and lean body mass, *N* = 90) calculated from total body water (TBW). Blubber thickness was measured from the body surface to the depth of the first evident muscle layer with the SonoSite 180PLUS ultrasound (SonoSite Inc., WA, USA) at a single site, dorsally, on the right flank.

Total body water was determined using the deuterium isotope (^2^H_2_O) dilution method. Blood samples to identify background isotope levels were collected either from the interdigital rear flipper vein or the caudal gluteal vein prior to the intermuscular administration of deuterium oxide (dose 0.5 g kg^−1^). Additional blood samples were collected during a subsequent anesthesia procedure at a minimum of 2 h after deuterium dosing to ensure equilibrium with body water (Rea unpublished data). Sera and reference samples were stored on dry ice in the field, and at –80 °C upon return to the lab. Samples were analyzed for TBW using infra-red (FTIR) spectrophotometry. Total body water was converted to total body lipid (TBL) mass using predictive equations derived for pinnipeds [33] and correcting for the approximate 4% overestimation of TBW [34], and then expressed as percentage of total mass (percent total body lipid, %TBL).

### Statistical Analysis

JMP (SAS Institute Inc., Cary, NC) statistical software was used for all summary statistical analyses. Paired *t*-tests were used to compare metabolic rates pre- and post-anesthesia. Differences in RMR between males and females were compared using two-sample *t*-tests. One-way analysis of variance (ANOVA) was employed to compare group means across age categories and Tukey-Kramer HSD test was used for post hoc pairwise comparisons. To control the cumulative Type I error rate, Bonferroni correction was applied to pairwise comparisons. All means are presented with ± standard error (SE). Results were considered significant at *P*<0.05.

In addition to the above summary statistical approach, we wished to develop insight on model structures rather than test existing hypotheses, so simply performing stepwise regression would not give us any information about model uncertainty when compared to other potential models. We also wished to avoid the problems inherent in stepwise procedures [35]–[37], specifically in our data set where variables of temperature, body mass and TBL tended to be confounded by location. In order to account for potential error in model selection, an information-theoretical approach which uses Akaike Information Criteria (AIC) as a metric to compare fit and hence potential suitability of the various candidate models was used in R (R Foundation for Statistical Computing, Vienna, Austria) to examine the utility of location, age, %TBL, ambient temperature and sex as predictive variables when modeling RMR [38], [39].

Metabolic rate was regressed on all 31 combinations of these 5 variables, while always including body mass^0.75^ as a predictor [40]. For example, the model including all variables was
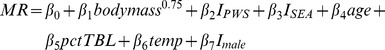



where 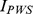, 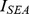 and 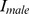 are indicators that equal 1 when the animal was from PWS, from SEA or was male respectively, 0 otherwise. Note that 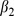 and 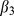 represent offsets from CAI for PWS and SEA respectively.

For each of the regressions the corrected Akaike information criterion (AICc), the difference with the lowest observed AICc (ΔAICc), and Akaike weights were calculated, and the models ranked based on the Akaike weights. The importance of each covariate was assessed by the sum of Akaike weights for all models that included that covariate. A model average was also calculated using the top 95% of the Akaike weights. This represents our “best” model that includes the variability of model selection.

## Results

### Metabolic Rate

Resting metabolic rate measured on a subset of sea lions (*N* = 19) showed no significant differences (*t* = 1.29, d.f. = 18, *P* = 0.214) between pre- and post-anesthesia measurements. Thus, isoflurane anesthesia exposure was not considered to be a factor influencing metabolic rate in this study. Milk was discovered in the stomach of a small number of animals (*N* = 14) ranging in age from 2-17 mo, with all but one animal being younger than 12 mo of age. No significant differences in RMR (all *P*>0.05) were detected between animals with and without milk according to age class and capture location, therefore, all sea lions (regardless of the presence of milk in the stomach) were included in the remainder of analyses.

**Figure 2:**
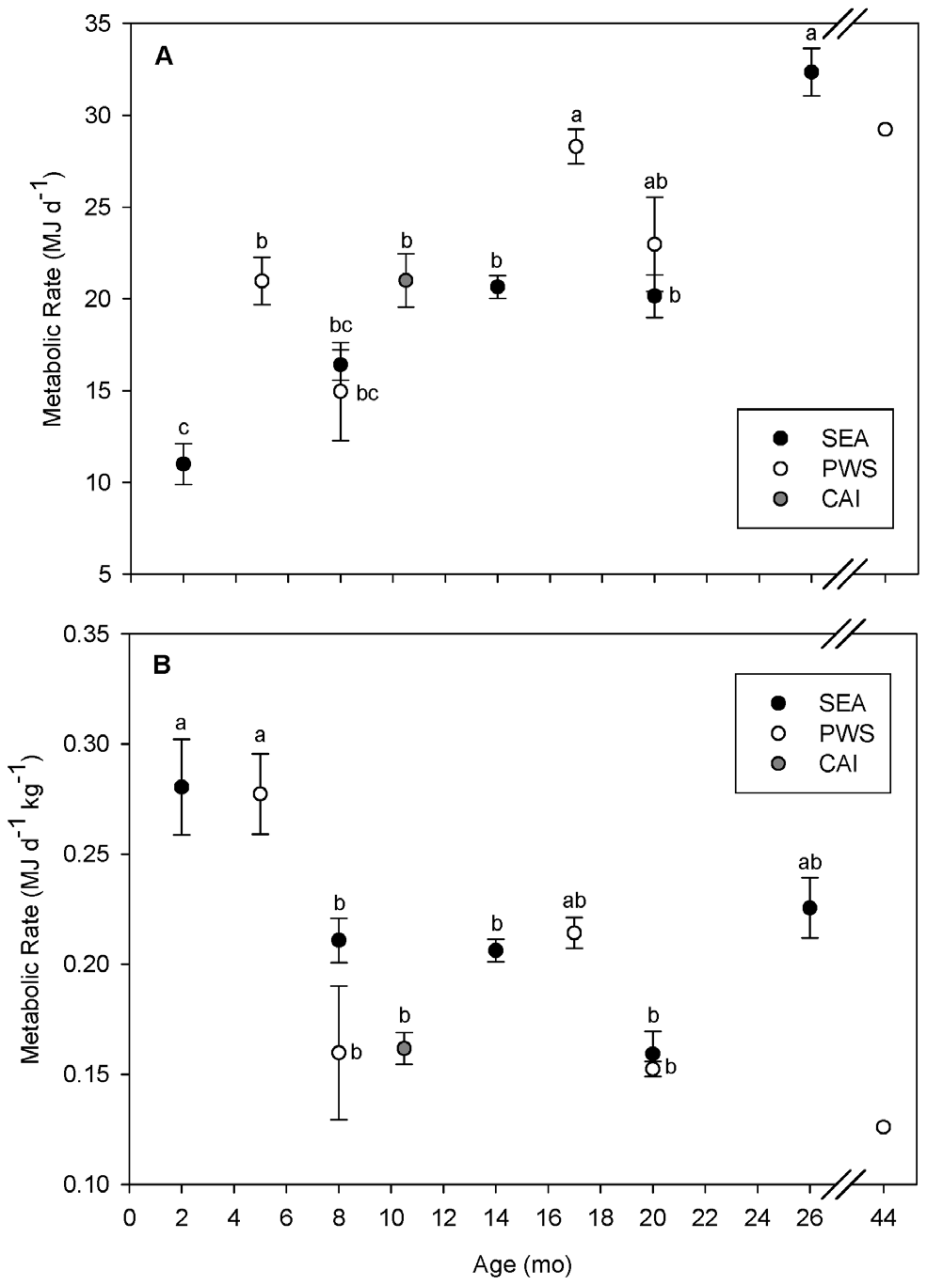
Mean metabolic rate (MJ d^−1^, ± SE) (A) and mean mass-specific metabolic rate (MJ d^−1^ kg^−1^, ± SEM ) (B) as a function of age (mo) for sea lions captured in Southeast Alaska (SEA), Prince William Sound (PWS), and the Central Aleutian Islands (CAI). Within each region of study, metabolic rate levels with similar letters showed no significant differences, while levels with differing letters were considered significantly different at *P*<0.05.

Resting metabolic rate in air ranged from 6.7 to 36.2 MJ d^−1^ and varied with sea lion age and body mass (Table 1). While younger sea lions were generally smaller and had lower absolute metabolic rates than older juveniles, several age classes displayed no differences in RMR (Figure 2a). Among the pups, 2 mo old animals from SEA (*N* = 9) had the lowest mean RMR and 10.5 mo old animals from CAI had the highest mean RMR (*N* = 15). Mass-specific RMR (MJ d^−1 ^kg^−1^) declined with increasing sea lion age (and size), with younger sea lions having significantly higher mass specific RMRs than older animals (Figure 2b).

Metabolic rates measured in individual sea lions in the present study ranged from 1.1 to 4.1 (overall mean 2.2 ± 0.1) times the predicted basal metabolic rate of similarly sized adult terrestrial mammals [40]. The mean value of 2.2 times predicted RMR for all animals was similar to predicted levels when sea lions were considered by age.

For their age, CAI pups were larger than similarly aged animals from the other capture locations and were of similar total mass to juvenile animals in SEA and PWS (Table 1). As a percentage of body mass, %TBL ranged from 3.5 to 40.5% in pups and 6.4 to 36.5% in juveniles and was highly variable within and between age categories and among regions of capture. There were no significant differences (*P*>0.05) in mean %TBL between male and female sea lions for all age categories in this study where sufficient sample sizes allowed comparisons. Pups from CAI had significantly greater amounts of total body lipid compared with all ages of sea lions from both PWS and SEA (all P<0.05). Regionally, sea lion pups captured in CAI also had the thickest mean blubber depths measured (2.2±0.1 cm).

Metabolic rate was measured in free-ranging sea lions with a range of ages and body condition, captured over varying ambient air temperatures (range –6.5 to 19.2°C) in three regions of Alaska. However, the only covariate besides body mass^0.75^ (that was designated to be in all models) with strong support from the data for inclusion in a predictive model was %TBL (Table 2) as it was included in all models in the top 95% of weight. Models including ambient air temperature represented 65% of the weight, implying some support. Location of capture showed inconclusive support with 57%, and age and sex with 26% and 24% respectively were not useful predictors. No single model was found to have strong evidence that it fit the data better than the other candidates, since the strongest was the model with body mass^0.75^, ambient temperature, and %TBL and it represented only 22% of all model weight (Table 3). Results from averaging the top 95% of potential models are shown in Table 4. Confidence intervals on the various coefficients indicate only body mass^0.75^ as having evidence of being different from zero. For example, for a 10% increase in mass we would expect RMR to increase 0.37 to 0.79MJ d^−1^. Despite %TBL being included in all relevant models (showing a negative association with RMR), it had relatively high SE so the overall model average indicates limited usefulness.

**Table 2:** Total model weight of variable inclusion for different exponents on body mass.

Variable	Body Mass^0.75^	Body Mass^0.67^
P, Total body lipid	1.0	1.0
T, Ambient temperature	0.65	0.63
L, Location	0.57	0.58
A, Age	0.26	0.25
S, Sex	0.24	0.24

**Table 3:** Candidate models using body mass^0.75^.

Model	AICc§	ΔAICc	Akaike Weight‡	Cumulative Weight
**TP** *	517.4	0.0	0.22	0.22
**LP**	517.8	0.4	0.18	0.40
**TLP**	518.3	0.9	0.14	0.55
**TPA**	519.3	1.9	0.08	0.63
**TPS**	519.7	2.3	0.07	0.70
**LPA**	520.1	2.6	0.06	0.76
**LPS**	520.1	2.7	0.06	0.82
**TLPA**	520.6	3.2	0.05	0.87
**TLPS**	520.6	3.2	0.05	0.91
**TPSA**	521.6	4.2	0.03	0.94
**LPSA**	522.4	5.0	0.02	0.96
P	522.6	5.2	0.02	0.97
TLPSA	522.9	5.5	0.01	0.99
PA	524.8	7.4	0.01	0.99
PS	524.8	7.4	0.01	1.00
PSA	527.0	9.6	0.00	1.00
L	655.4	138.0	0.00	1.00
TL	656.6	139.2	0.00	1.00
LA	657.5	140.1	0.00	1.00
LS	657.6	140.2	0.00	1.00
TLA	658.7	141.3	0.00	1.00
TLS	658.9	141.5	0.00	1.00
LSA	659.7	142.3	0.00	1.00
TLSA	660.8	143.4	0.00	1.00
TA	662.7	145.3	0.00	1.00
TSA	663.6	146.2	0.00	1.00
T	664.2	146.8	0.00	1.00
TS	665.9	148.5	0.00	1.00
A	669.1	151.7	0.00	1.00
S	669.7	152.3	0.00	1.00
SA	670.8	153.4	0.00	1.00

Models in bold were used for coefficient averaging.

The corrected Akaike information criterion (AICc) was used to rank the models using the difference with the lowest observed AICc (e.g., ΔAICc).

Akaike weights represent the probability that a given model best reduced the information loss for the observed data.

T, Ambient temperature; P Total body lipid (%); L, Location; A, Age; S, Sex.

**Table 4:** Averaged coefficients for top 95% of model weights.

	Coefficient	SE	Adjusted SE*	Lower CI§	Upper CI
Intercept	4.605183	4.260	4.297	–3.817	13.028
Body mass^0.75^	0.535295	0.099	0.100	0.340	0.731
Ambient temperature	–0.09227	0.121	0.121	–0.330	0.146
Location PWS	3.003093	2.123	2.138	–1.188	7.194
Location SEA	1.760194	1.746	1.766	–1.700	5.221
Total body lipid (%)	–0.10581	0.075	0.076	–0.254	0.043
Sex M	–0.03401	0.577	0.586	–1.182	1.114
Age in months	–0.00621	0.096	0.098	–0.198	0.185

Adjusted SE includes model selection uncertainty (Burnham and Anderson 2002).

The upper and lower endpoints represent those of a 95% confidence interval.

SE, standard error; CI, confidence interval.

For comparison with these results, we also ran the same model selection, but used 0.67 as the coefficient on body mass rather than 0.75 (Table 2). While the specific ordering of the models changed, the general ordering was still very similar and %TBL was still the only other covariate apart from body mass^0.67^ that showed strong support. One reason for this was that over the range of masses we observed, small changes to the exponent on mass differed approximately by a multiplicative constant, which could easily be accounted for in the associated regression coefficient.

Male sea lions were heavier than females and were significantly longer in dorsal standard length and larger in all girth measurements for a given age (all *P* <0.05). Although this species is sexually dimorphic from birth, gender differences in RMR were not supported by this model.

## Discussion

Under short-term conditions of deceased energy intake sea lions would be expected to increase foraging efforts since further expenditure of energy is likely off-set by a reasonable expectation of success during the foraging trip. However, when faced with predictable or large-scale shortages of energy intake, physiological adaptations that would limit energy expenditures include a reduction in activity, thermoregulation, and metabolism. These strategies serve to increase survival time by limiting the loss of body mass. Animals undergoing periods of reduced energy intake would also be expected to be in ‘poorer’ body condition than animals with continuous access to food. These animals should have reduced blubber and lipid stores due to catabolism of these tissues for fuel. If sea lions from the western declining population were experiencing chronic periods of reduced food intake during this study, we should see evidence of this in measures of body composition and RMR.

The information-theoretical modeling approach employed in this study did not find evidence that any variable (%TBL, ambient temperature, age, sex or location), other than body mass^0.75^ played a strong role in determining the RMR of pups and juvenile Steller sea lions in Alaska. Although %TBL was included as a variable in all models accounting for 95% of the weight of all models considered, and showed the expected negative relationship with RMR (increasing %TBL reflects a relatively smaller metabolically active lean body proportion), large confidence intervals precluded the usefulness of this variable to predict RMR. Location and ambient temperature were included in only the top 57 to 63% of the weight of models, suggesting that they have less influence on the relationship of RMR among sea lions than %TBL, and that age and sex of the individual did not figure strongly into the relationship or these effects were already accounted for by the body mass^0.75^ variable. With inconclusive support of regional RMR differences, along with evidence of higher body mass and %TBL at age for pups in the western DPS, and the CAI specifically, we conclude that during this study sea lions were acquiring adequate energy intake and there was no evidence of metabolic depression in the western DPS. We conclude that any tendency for CAI pups to exhibit slightly lower RMR can be attributed to the relatively lower proportion of metabolically active lean body mass compared to their eastern DPS counterparts.

While evidence of widespread metabolic depression was not evident in sea lions from the western population during this study, it is difficult to predict whether the metabolic response might be greater at times of year when sea lions are more likely to experience decreased energy intakes (e.g., winter) or in conjunction with life history events that are energetically expensive (e.g. molting). Although sampling trips were conducted at various times of year, small sample sizes and non-similar age classes between locations prevented comparisons of RMR by season. Food restriction experiments with arctic foxes revealed no effect of season on the degree of metabolic depression [41], however, short-term reductions in energy intake over the course of a year revealed seasonally dependent changes in metabolism, body composition and body mass in captive Steller sea lions [42]. Sea lions responded differently to reduced food intakes depending on season and differences in body mass loss based on diet type (high lipid or low lipid) suggested that diet composition may pose an additional impact during certain times of the year [42]. Steller sea lions undergo their annual molt in the fall (Aug/Sept), when it has been suggested that metabolism would be elevated due to new hair growth and/or thermoregulatory costs. Metabolic studies in otariids have shown that pups have elevated metabolic rates during molt [43], [44], but this relationship has not been explored in juveniles or adults. Sampling of sea lions during the molt was avoided during this study to facilitate a concurrent study that relied upon attachment of dive telemetry instruments to the pelage and to avoid any potential confounding effects on resting metabolism. Further studies looking at seasonal changes in metabolism would be valuable to making better predictions about energetic requirements.

Other studies have tried to identify differences in energy metabolism indirectly through the measurement of thyroid hormones. In mammals, triiodothyronine (T_3_) and thyroxine (T_4_) thyroid hormones are essential for growth, relate directly with energy intake and indicate a continuous ongoing regulation of metabolism in relation to caloric supply. Myers et al. [45] noted regional differences in thyroid concentrations measured in Steller sea lions. They found that pups from SEA had the lower concentrations of thyroid hormones compared to western regions of Alaska and Russia and suggested that elevated thyroid levels were due to an increased metabolic demand to maintain thermal homeostasis. We believe that these lower hormone levels in SEA could also result from lower growth rates seen in SEA pups compared to other regions. Additionally, while a correlation between changes in thyroid hormones and metabolic rate has been measured in pinnipeds [46], [47], controlled feeding studies with captive juvenile Steller sea lions undergoing a 20–30% reduction in intake failed to correlate changes in thyroid levels to decreased measurements of daily metabolic rate [48], [49].

Juvenile Steller sea lions in this study were reported to have mean RMRs on average 2.2 times (range 1.1–4.1 times Kleiber), the metabolic rate predicted by Kleiber [40] for an adult mammal of similar body size. This is consistent with elevated metabolic rates noted in other juvenile otariids [44], [50], [51].

Male sea lion pups were 15 to 27% heavier than female sea lions (depending on age) in this study. Sexual dimorphism is a characteristic of all otariids and its influence is evident early in development where, maternal investment during gestation, average birth mass, mass at weaning, and growth rates of male pups are generally greater than those of females [52], [53]. This disparity in size is evident in neonate Steller sea lion pups [54], and several other young otariids (e.g., [55]–[57]). While a direct comparison of size based on age between all three regions was not feasible due to the small sample size of this study, size differences have been documented among these capture regions (Rea unpublished data). Steller sea lion pups from SEA were smaller in total body mass at similar ages compared with sea lions from PWS and CAI. This pattern of increasing body mass from east to west along with the larger body size and fat content of CAI sea lions has been observed in other studies [54], [58], [59] and is contrary to observations of nutritionally compromised animals being smaller and thinner (e.g., [60], [61]).

As a percentage of body mass, %TBL ranged from 2.8 to 35.3% in pups and from 6.9 to 32.7% in juveniles in this study and was highly variable within and between age categories and locations. Average %TBL in 2 mo old sea lions was low (9% of total body mass), only 3% higher than levels measured at birth [54], reflecting the fact that Steller sea lion pups are born with small energy reserves and exposure to short-term fasting during periods of maternal foraging results in slower gains in lipid mass compared to phocid seals. Additionally, for their large size, 2 mo old Steller sea lions had much lower mean %TBL compared to smaller otariid species [44], [62], [50]. This difference is largely a reflection of life history strategy since most of these species typically undergo weaning earlier than Steller sea lions.

Sea lions from CAI were larger and fatter than sea lions from PWS and SEA when differences in age are accounted for. It is difficult to speculate on why CAI sea lions are so much bigger than their PWS and SEA cohorts given the single age class of animals sampled from this location in the present study. Other studies have suggested that sea lions are smaller and hence, thinner, in SEA due to density-limiting factors. Pups from SEA have also been shown to have slower growth rates and face longer fasts due to shorter maternal attendance [58]. It is also possible that seasonal fluctuations in body fat stores may be driving this difference. Captive Steller sea lions were shown to have increasing body fat levels in the spring (the same time during which sea lion captures in the CAI took place in this study) and decreasing body fat stores into fall [42]. Additional collections of sea lion of varying ages from CAI would help clarify the discrepancy in size and body fat in CAI animals.

Steller sea lions are considered to be relatively ‘lean’ animals, with thin blubber layers [63]. Blubber depths measured in this study were comparable to depths measured in captive-held Steller sea lion juveniles [64], [65]. It should be noted that while blubber depth was only measured at one location along the body trunk (dorsal hip region), this is an area where seasonal changes in the blubber layer, in parallel with mass changes, would be evident [65].

Aerial, land, and ship-based surveys of pup and non-pup Steller sea lion counts conducted during the time frame of this study showed a trend of slowing population decline in some regions of the western DPS. Non-pup sea lions counts in the western stock were found to increase 11–12% between 2000 and 2004, with the estimated annual rate of change of +2.9%. Although increases were noted during this period of time, they were not uniform across the entire western range of rookeries, and populations in the eastern and the central Gulf of Alaska and the western Aleutian Islands declined considerably. A similar pattern was seen in pup counts in this region [23], [24]. Western DPS sea lions in this study were captured from Prince William Sound west through the Central Aleutian Islands, and sampling sites included a number of rookeries/haul outs which were undergoing decline during this period. Taken together, this data suggests that if nutritional stress were impacting the energetic intake of Steller sea lions in western Alaska, the effects were not limiting enough to induce metabolic depression. This is consistent with more recent studies that have suggested the continued decline of western Steller sea lions populations may be attributed to other anthropogenic causes (e.g., [26], [66]).

This is the first study to measure RMR in free-ranging juvenile Steller sea lions. To our knowledge, no studies have sought to measure metabolic depression as a response to prey limitations in free-ranging marine mammals. Understanding basic physiological parameters such as metabolic rate has important implications to understanding diving limitations and capabilities, maintenance requirements for energy budgets, thermoregulatory constraints, and bioenergetics modeling of complicated physiological systems.
